# Molecular Lego of Human Cytochrome P450: The Key Role of Heme Domain Flexibility for the Activity of the Chimeric Proteins

**DOI:** 10.3390/ijms23073618

**Published:** 2022-03-25

**Authors:** Gianluca Catucci, Alberto Ciaramella, Giovanna Di Nardo, Chao Zhang, Silvia Castrignanò, Gianfranco Gilardi

**Affiliations:** Department of Life Sciences and Systems Biology, University of Torino, 10123 Turin, Italy; gianluca.catucci@unito.it (G.C.); alberto.ciaramella@unito.it (A.C.); giovanna.dinardo@unito.it (G.D.N.); chao.zhang@unito.it (C.Z.); silvia.castrignano@unito.it (S.C.)

**Keywords:** aromatase, 3A4, BMR, stability, flexibility, chimerization

## Abstract

The cytochrome P450 superfamily are heme-thiolate enzymes able to carry out monooxygenase reactions. Several studies have demonstrated the feasibility of using a soluble bacterial reductase from *Bacillus megaterium*, BMR, as an artificial electron transfer partner fused to the human P450 domain in a single polypeptide chain in an approach known as ‘molecular Lego’. The 3A4-BMR chimera has been deeply characterized biochemically for its activity, coupling efficiency, and flexibility by many different biophysical techniques leading to the conclusion that an extension of five glycines in the loop that connects the two domains improves all the catalytic parameters due to improved flexibility of the system. In this work, we extend the characterization of 3A4-BMR chimeras using differential scanning calorimetry to evaluate stabilizing role of BMR. We apply the ‘molecular Lego’ approach also to CYP19A1 (aromatase) and the data show that the activity of the chimeras is very low (<0.003 min^−1^) for all the constructs tested with a different linker loop length: ARO-BMR, ARO-BMR-3GLY, and ARO-BMR-5GLY. Nevertheless, the fusion to BMR shows a remarkable effect on thermal stability studied by differential scanning calorimetry as indicated by the increase in T_onset_ by 10 °C and the presence of a cooperative unfolding process driven by the BMR protein domain. Previously characterized 3A4-BMR constructs show the same behavior of ARO-BMR constructs in terms of thermal stabilization but a higher activity as a function of the loop length. A comparison of the ARO-BMR system to 3A4-BMR indicates that the design of each P450-BMR chimera should be carefully evaluated not only in terms of electron transfer, but also for the biophysical constraints that cannot always be overcome by chimerization.

## 1. Introduction

The cytochromes P450 superfamily are heme-thiolate monooxygenases that are able to perform a high variety of regio- and stereo-selective reactions [1,2,3,4,5,6]. In order to carry out their reaction, they require fine coupling to a reductase [7]. The overall process architecture is designed for coupling a two-electron delivery from the reducing agent NADPH to a one-electron acceptor, ferric P450. However, the electron transfer process can be different depending on the redox partner involved. In the mammalian cytochrome P450 reductase (CPR), two electrons from NADPH enter the enzyme as a hydride ion to the FAD, followed by intramolecular electron transfer to FMN that shuttle between the semiquinone and hydroquinone states [8]. In the case of the bacterial system cytochrome P450 BM3, the P450 heme is reduced by the semiquinone of FMN rather than by FMNH_2_ of BMR [9,10]. The electrons are key for the reaction of the enzyme, as they modify the oxidation state of the heme iron required for dioxygen activation, with the formation of the Compound I key to the monooxygenation of the target substrate. During this process, it is very well known that uncoupling reactions may occur, and these unproductive routes may result in products of various oxygen containing species that do not involve the target substrate. One the expected undesired products is hydrogen peroxide [11,12,13]. The route leading to hydrogen peroxide, the “peroxide shunt”, is reversible, and therefore—in some P450s—hydrogen peroxide can be exploited to drive the monooxygenation of the target substrate, circumventing the addition of a reductase, NAD(P)H and molecular oxygen. This is the case of the cytochrome P450 peroxygenases that can operate also without reductase but with the addition of peroxide as source of oxygen and reducing equivalents [14,15,16,17,18,19].

Nature has evolved several different P450 classes on the basis of their electron transfer requirements. Ten different combinations of reductase systems-P450 have been classified. Enzymes belonging to class I to VI require a separate reductase that must interact using a specific mechanism of protein–protein interaction. On the other hand, classes from VII to X are actually self-sufficient [20,21,22], meaning that they are composed by a single polypeptide chain that includes a reductase and P450 domain [23]. As for the P450 of Class II, cytochrome P450 reductase (CPR) is the only physiological partner for many different P450s and how CPR is able to mediate electron transfer to a variety of different electron acceptors remains an open question [8]. Over the years, researchers have invested considerable effort in understanding the electron transfer mechanism between the CPR and the P450s, and they also have provided biotechnological solutions to sustain the activity of P450s [24,25,26,27,28,29]. Among these, the so-called *“Molecular Lego”* was introduced in 1998 when it was demonstrated that non-physiological electron transfer fused at genetic level in a single polypeptide chain can provide self-sufficient engineered P450 catalysts [30]. Initially, in order to address the solubility problem of the human P450 enzymes, the soluble reductase domain of *Bacillus megaterium* (BMR), a NADPH-dependent oxidoreductase composed by FAD and FMN subdomains, was fused in a single polypeptide chain to an N-terminally engineered CYP2E1 via a Pro-Arg-Ser connecting loop. In this case, the data showed how the 2E1-BMR chimera retains the characteristic activities of native 2E1 and it became catalytically self-sufficient requiring only external NADPH as an electron source for catalysis [31]. Among all the CYP isoforms relevant to drug metabolism, CYP3A4 has been the election model system for studying the biochemistry involved in the molecular Lego approach. Early characterization work showed that the chimera between the CYP3A4 and the BMR, analyzed by far-UV circular dichroism, preserved all the secondary structure elements expected from the structure of the isolated domains. Furthermore, chemical denaturation of the 3A4-BMR chimera identified the clear presence of two domains that undergo separate transitions upon unfolding [32]. In terms of functionality, addition of detergent or increased enzyme concentration had no influence on the activity [32]. Experiments of 3A4-BMR reconstitution could establish the best reaction conditions for determining coupling efficiency [33] (the amount of NADPH used to perform the desired catalytic activity), leading to the design of different loops connecting the two domains [34]. Two new chimeras were designed that extended the length of the Pro-Arg-Ser loop with the addition of three or five glycines, resulting in the 3A4-BMR-3GLY and 3A4-BMR-5GLY chimeras [34]. All the 3A4-BMR chimeric proteins were found to be significantly faster than 3A4-CPR couple at oxidizing NADPH both in the absence or presence of substrate [34]. The BMR domain was found to strongly stabilize the heme domain within the chimera as demonstrated by kinetics of carbon monoxide decay [34]. In terms of loop length when the three different chimeras are compared, the 3A4-BMR-5GLY one showed a higher stability of the P450 module, a higher coupling efficiency, and also a higher cytochrome c reduction rate [34]. The loop length also positively impacted the binding affinity and cooperativity for testosterone ultimately leading to increased k_cat_ [35]. Furthermore, hydrogen/deuterium exchange kinetics measured by FTIR revealed that 3A4-BMR-5GLY displayed higher exposure of the protons indicating that the surface area between the P450 and the BMR domain is more accessible to the solvent [36]. Finally, electrochemistry experiments performed in different cross-linking preparations showed that a more flexible immobilizing gel results in higher electrocatalysis, especially for the 3A4-BMR-5GLY chimera [36]. All the above mentioned data indicate that a connecting loop increased by five glycine residues is able to improve the stability of the protein and the coupling efficiency through a higher flexibility of the system [34,35,36]. Moreover, molecular modelling studies indicated that the chimeras between the CYP3A4 and BMR, the length of the loop is crucial for the overall flexibility of the system because a shorter loop is predicted to form a secondary structure that rigidifies the movement of the FMN domain in the process of docking to the heme proximal site of the P450 domain [35].

In this work, we integrate previously obtained data with a biophysical characterization of the chimeras between the CYP3A4 and BMR using differential scanning calorimetry. In addition to 3A4 chimeras, we also tested three different aromatase-BMR constructs (ARO-BMR, ARO-BMR-3GLY, and ARO-BMR-5GLY). As for the corresponding 3A4-BMR chimeras, each construct differs only for the length of the loop that contains either zero, three, or five glycines. For ARO-BMR chimeras, since they are produced for the first time, pure proteins were tested for their activity using androstenedione as marker substrate. We then compare the unfolding process of the isolated domains to the chimeras bearing either zero, three, or five glycines in the linking loop. By comparing purified, isolated BMR, ARO, and 3A4 to the corresponding the chimeras, we are able to perform deconvolution of each species from the chimeras. Peaks assignment allows the determination of the T_m_ and the enthalpy associated to each transition and appreciate the stabilizing role of BMR. Finally, we interpret the results obtained on 3A4 and ARO chimeras on the basis of a model that shows the potential of the molecular Lego approach as well as its current limits.

## 2. Results

### 2.1. Characterization and Activity of the ARO-BMR Chimeras

**Figure 1:**
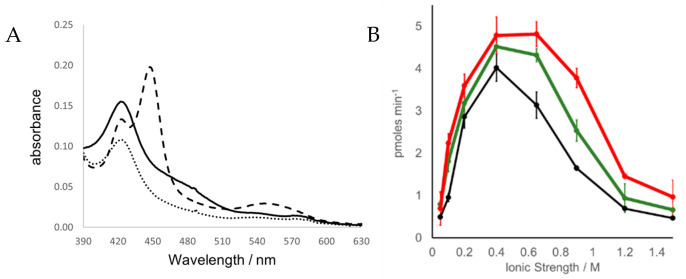
(**A**) CO binding assays of ARO-BMR chimeras. Oxidized, reduced, and CO bound spectra are shown in solid, dotted, and dashed lines, respectively for ARO-BMR. (**B**) Ionic strength dependence of ARO-BMR chimeras’ activity. Activities of ARO-BMR, ARO-BMR-3GLY, and ARO-BMR-5GLY are reported in black, green, and red respectively.

Three ARO-BMR chimeras were engineered to differ by the length of the loops connecting the ARO domain with the BMR domain, namely a short Pro-Arg-Ser loop (ARO-BMR), a medium loop with an additional three Gly (ARO-BMR-3GLY), and a longer loop with five Gly (ARO-BMR-5GLY). All proteins were successfully expressed in *E. coli* and purified using two chromatographic steps [32,35]. In all cases the final yield was between 6 and 10 mg/L of culture. Spectrophotometric characterization was employed to record the behavior of the chimeras in the oxidized, reduced, and carbon monoxide-bound forms. The UV–visible spectra of the three proteins show a Soret peak at 420 nm, the β and α bands at 535 and 570 nm and the broad shoulder at 450–475 nm that indicate the incorporation of the flavin cofactors in the oxidized form in the BMR domain (Figure 1).

For the isolated (non-chimeric) aromatase, the Soret peak is found at around 418 nm [37], while the shift to 420 nm is observed in all the chimeras. In the absence of a Type II ligand shift, the maximum at 420 nm evidences the presence of a partial transition from P450 to the inactive P420 state [38,39,40,41,42]. As a preliminary experiment, we tested the ability of the ARO-BMR to use NADPH as reductant and BMR as reductase in a stopped-flow experiment as previously reported by our group for 3A4-BMR [35], but we could not observe either reduction in ARO or a CO binding (Figure S1). In order to provide more evidence to the hypothesis that the heme domain within ARO-BMR is the sole responsible of the diminished activity of the chimera, we performed a new set of experiments in which we have investigated the reducibility of FAD of BMR by NADPH. The experiments were aimed at understanding whether the BMR domain within ARO-BMR differed from isolated BMR. Therefore, we carried out rapid kinetics experiments on both ARO-BMR and BMR purified proteins using either a 10-fold excess or equimolar NADPH in the absence of oxygen as previously done [10]. The data reported in Figure S2 clearly show that when either BMR or ARO-BMR is rapidly mixed with a 10-fold excess of NADPH, both proteins are rapidly reduced as indicated by the FAD reduction rates: 140.23 ± 8.37 s^−1^ for ARO-BMR and 154.87 ± 12.15 s^−1^ for BMR. The high level of comparability in terms of reduction rate clearly demonstrates that the BMR domain within the ARO-BMR chimera behaves like the isolated BMR. Furthermore, when ARO-BMR is rapidly mixed with equimolar NADPH, we were able to monitor all the expected flavin species (Figure S3). As shown in Figure 1, the addition of excess sodium dithionite to ARO-BMR chimeras caused the reduction in both the flavins and heme iron as demonstrated by the decrease in the intensity of the bands at 420 and 475 nm. Addition of carbon monoxide to the reduced chimeras caused a clear shift to 450 nm, indicating the presence of a correct heme geometry in the P450 domain (Figure 1A).

None of the ARO-BMR proteins exhibited a full shift to 450 nm and a clear trend is present among the three different chimeras indicating that ARO-BMR has the highest content of active protein followed by ARO-BMR-3GLY and ARO-BMR-5GLY (data not shown). This trend was not expected because previous work on 3A4-BMR chimeras showed that there is no direct correlation between the length of the loop and the folding of active protein [34]. However, as noticed before, the proteins show a Soret peak at 420 nm indicating the presence of a partial transition from P450 to the inactive P420 state. These data indicate that BMR affects the heme properties of the three proteins, with the construct carrying five glycines showing the highest one, indicating a different type of interaction between the BMR and ARO domains.

Substrate binding experiments studied were performed by UV–vis spectroscopy using androstenedione. Only a small spectral transition of the Soret peak from 420 to 394 nm was observed for all the three chimeric proteins (Figure S6), whereas previously published data with ARO have shown an almost full low-to-high spin transition was obtained in the same conditions [37,43].

The activity of the chimera was then assayed to study the effect of chimerization with a non-physiological redox partner on the enzyme activity. Reactions were carried out for 30 min at 30 °C using NADPH as electron donor and saturating amounts of androstenedione as substrate. They were heat inactivated for 10 min at 90 °C and centrifuged for 5 min at 11,000× *g*. The estrone product was quantified using a direct competitive ELISA [44,45], which allowed its detection within the range from 25 to 350 pg/mL (from 0.1 to 1.3 nM). The turnover numbers are reported in Table 1 where the activity of the non-chimeric enzyme is reported when working with its physiological electron donor CPR.

The activity data show that chimerization dramatically affects the activity of aromatase. Indeed, ARO-BMR chimeras all show a 10^3^ fold decrease in activity when compared to the concerted catalysis of ARO and CPR in solution. Moreover, the length of the loop does not seem to significantly impact the activity of the enzyme. For comparison, the activity of the corresponding 3A4-BMR fusions was previously reported and the chimeras showed a 20-fold loss in activity when compared to the isolated physiological partners, even if the coupling efficiency was significantly improved [34]. In this case, an effect of the loop length was observed with the construct carrying the longest loop being the one with the highest activity and coupling efficiency. Since 3A4-BMR chimeras were previously found to exhibit the maximum activity at low ionic strength, indicating that charge–charge interactions could stabilize the complex [33], a similar experiment was carried out on ARO-BMR fusions. Thus, the activity was assayed at different ionic strength by measuring estrone formation maintaining the pH constant (pH 7.0). Figure 1B shows a peak in the activity at the ionic strength of 0.4 M for all the chimeras and, interestingly, ARO-BMR-5GLY activity is still at maximum even at higher ionic strength (up to 0.6 M), indicating a possible effect of the linker on the interaction between the two domains.

### 2.2. Differential Scanning Calorimetry

In order to understand how the stability of P450-BMR chimeras affects the functionality of the P450 enzyme, we conducted differential scanning calorimetry (DSC) experiments for both the single domains and the chimeric proteins.

**Figure 2:**
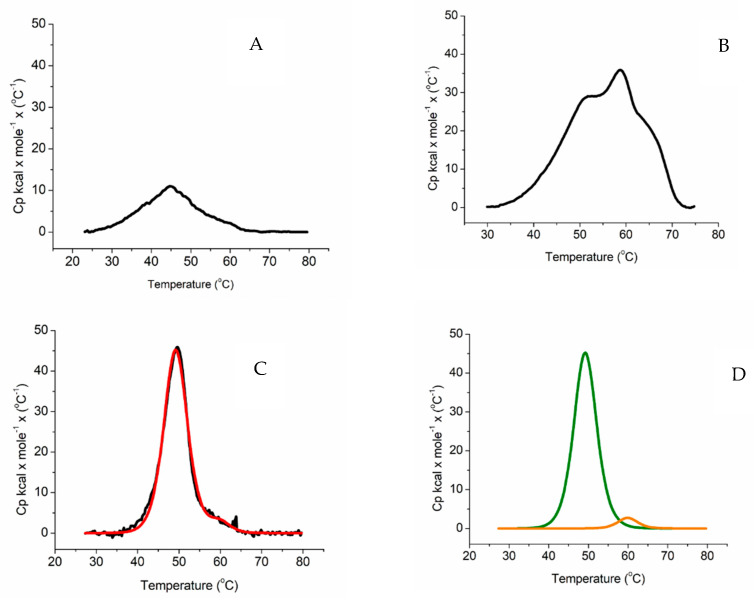
Thermograms of (**A**) aromatase, (**B**) CYP3A4, and (**C**) BMR. All measurements were carried out at scan/rate of 90 °C/h. Black lines are the experimental curves, the red line is the fitting of the experimental curve applying a non-two-state denaturation model. (**D**) Deconvolution BMR. The green line is the deconvolution of the first peak and the orange line is the deconvolution of the second peak.

Figure 2A shows that isolated aromatase exhibits a broad transition and low energy barrier to unfolding. The profile is consistent with the fact that aromatase is known to be unstable in the absence of a substrate or an inhibitor [47]. Indeed, it was previously reported that marginally stable proteins tend to follow a poorly cooperative folding process involving the formation of different populations of partly folded forms that are typically characterized by a low energy barrier to unfolding [48].

As a term of comparison, DSC was also carried out on the CYP3A4 alone. As shown in Figure 2B, also in the case of CYP3A4 the unfolding shows a poorly cooperative transition that is symptomatic of a low energy barrier to unfolding and unstable molecule that implies the presence of several protein populations. The data are in line with previous reports on this protein that was found to be aggregated with a T_m_ of 52.6 °C and a T_onset_ > 30 °C [49]. When compared to ARO, CYP3A4 shows a slightly higher T_onset_ because it starts denaturing above 30 °C.

On the other hand, BMR (Figure 2C) shows a high energy barrier to unfolding, indicating that this molecule is more stable in solution.

A key indicator for the stability of a protein is defined as the T_onset_ that is generally defined as the intersection of the tangents of the peak with the extrapolated baseline. Clearly, ARO displays a low T_onset_ because its denaturation process starts above 25 °C (Figure 2A). BMR has a higher T_onset_ because it starts denaturing > 35 °C (Figure 2C). Moreover, ARO wide transition is indicative of a heterogeneous protein population that is not representative of a well-defined fold. In the case of BMR, two clear peaks are present (Figure 2C–D). Since BMR is made of the two FAD and the FMN domains, the peak exhibiting higher enthalpy was assigned to the larger FAD domain and the remainder to the smaller FMN one (Figure 2C–D).

**Figure 3:**
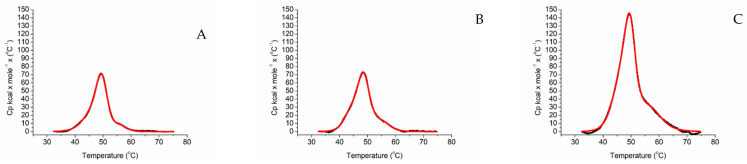

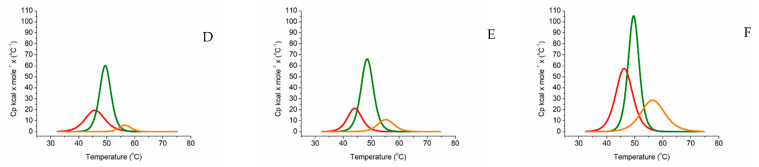
Thermal unfolding measured by DSC of ARO-BMR chimeras. Thermograms of (**A**) ARO-BMR, (**B**) ARO-BMR-3GLY, (**C**) ARO-BMR-5GLY. All experiments were carried out at a scan/rate of 90 °C/h. The black lines are the experimental curve of the chimeras, the red lines are the result of the fitting applying a non-two-state denaturation model. Deconvolution of ARO-BMR (**D**), ARO-BMR-3GLY (**E**), ARO-BMR-5GLY (**F**). The red lines represent the deconvolution of the denaturation curve of aromatase, the green lines represent the deconvolution of the FAD subdomain from BMR, and the orange lines represent the deconvolution of the FMN subdomain from BMR.

The thermograms of the ARO-BMR chimeras are reported in Figure 3. They all show a cooperative unfolding process, and have a high energy barrier to denaturation, with ARO-BMR-5GLY showing the highest enthalpy (Figure 3C).

**Figure 4:**
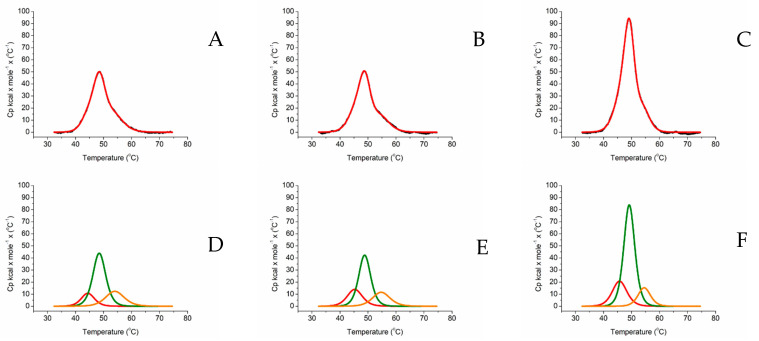
Thermal unfolding measured by DSC of 3A4-BMR chimeras. Thermograms of (**A**) 3A4-BMR, (**B**) 3A4-BMR-3GLY, (**C**) 3A4-BMR-5GLY. All experiments were carried out at a scan/rate of 90 °C/h. The black lines are the experimental curve of the chimeras, the reds line are the result of the fitting applying a non-two-state denaturation model. Deconvolution of 3A4-BMR (**D**), 3A4-BMR-3GLY (**E**), 3A4-BMR-5GLY (**F**). The red lines represent the deconvolution of the denaturation curve of CYP3A4, the green lines represent the deconvolution of the FAD subdomain from BMR, and the orange lines represent the deconvolution of the FMN subdomain from BMR.

Analysis of the thermogram going from the lower to the higher temperatures, and comparing the profiles obtained from the separate proteins, it was possible to assign the first peak to aromatase [47], the second one to the FAD domain of BMR and the third to its FMN domain (Figure 3D–F).The assignment of the peaks is strongly influenced by the cooperative behavior of BMR that is highly conserved in the isolated and chimeric proteins (Figure 2C and Figure 3A–C). Therefore, deconvolution analysis indicates the presence of three peaks corresponds to three protein domains (Figure 3D–F). However, when thermal unfolding experiments were carried out on the 3A4 chimeras, all three proteins showed a high energy barrier to denaturation and again 3A4-BMR-5GLY shows the highest enthalpy, following the same pattern observed for the ARO-BMR chimeras (Figure 4A–C). These data clearly show a stabilization effect driven by the fusion protein that is responsible for a cooperative unfolding process.

Peaks assignment, guided mainly by BMR (Figure 2C), attributes the first peak to the CYP3A4 domain, the second peak to the FAD domain of BMR, and the third peak to the FMN domain of BMR (Figure 4D–F).

All the thermodynamic parameters obtained from the analysis of the thermograms are reported in Table 2. The ARO chimeras show an overall conserved unfolding process that is very similar in terms of T_m_, but they exhibit significant changes in terms of enthalpy. For 3A4-BMR, there is a consistent increase in enthalpy with increasing linker length, whereas the enthalpy associated to the FAD domain decreases in 3A4-BMR-3GLY, but it increases again for 3A4-BMR-5GLY. The enthalpic contribution of the FMN shows a consistent decrease in enthalpy with increasing linker length. In the case of ARO-BMR the length of the linker increases all the enthalpic components except in the case of ARO-BMR-3GLY where the heme domain shows a decreased enthalpy when compared to ARO-BMR. In general, the extent of ΔH enhancement between the short linker and the 5GLY variant is greater in the case of ARO-BMR/heme vs. ARO-BMR-5GLY/heme than in the case of 3A4-BMR/heme vs. 3A4-BMR-5GLY/heme (Table 2).

## 3. Discussion

Cytochrome P450 enzymes are strictly dependent on the docking of a reductase partner able to deliver the electrons required for catalysis. Human class II P450s and their reductase CPR are both bound to the membranes of the endoplasmic reticulum. This poses challenges for their solubilization that is usually accompanied by instability and loss of activity [50], often with difficulties in the electron-transfer process and the increased degree of uncoupling and the production of reactive oxygen species [51,52].

A protein engineering approach, molecular Lego, has proved to be a successful strategy demonstrating that it is solves a series of problems such as protein solubilization and redox coupling. Literature data demonstrated the viability of this approach, particularly when P450 characterized by large substrate binding sites and flexible structures are employed.

**Figure 5:**
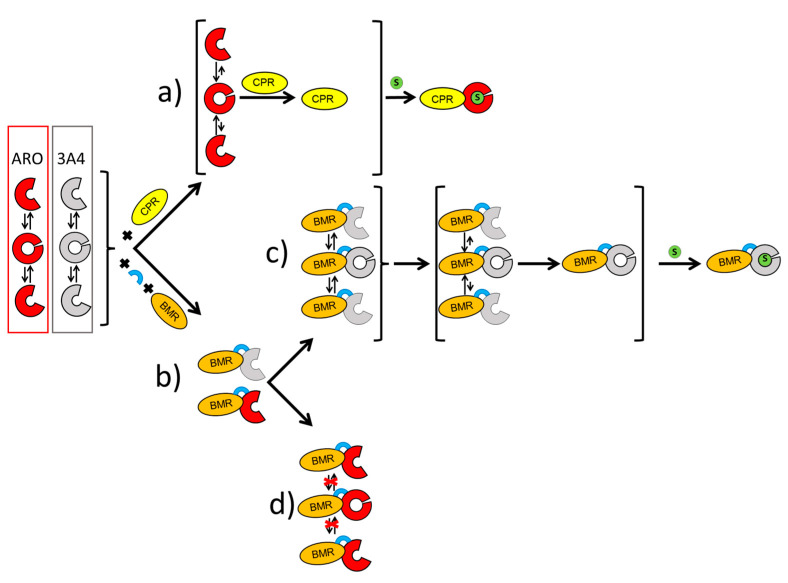
Effect of the molecular Lego on different CYPs P450. (**a**) Aromatase explores different conformational states and CPR is a positive effector in facilitating the pursuit of the optimal conformation for substrate binding [45]. (**b**) 3A4-BMR or ARO-BMR chimeras. (**c**) 3A4-BMR is able to perform the conformational transitions required for optimal substrate binding (**d**) BMR, although interacting with ARO as demonstrated by DSC data, is not able to allow the required conformational transitions that are necessary for optimal substrate binding in ARO.

In this work, we have challenged the molecular Lego constructing chimeras using the compact human aromatase, known to have a rather compact active site, with the bacterial reductase domain BMR. The activity and stability of the constructs were investigated to look for the key requirements needed for using this engineering approach. In terms of activity, we show that the chimerization process does not give a positive contribution to aromatase. Indeed, CO binding assays and estrone formation show that the BMR fusions negatively impact the activity the enzyme (Figure 1A, Table 1). Furthermore, the length of the loop connecting the BMR domain to the P450 domain does not seem to improve the electron transfer (Table 1). Interestingly, ARO-BMR-5GLY activity shows resistance to high ionic strengths indicating a possible increased stability of this chimera (Figure 1B). On the other end, differential scanning calorimetry clearly showed how the BMR domain is extremely important for the stabilization of the aromatase domain. The chimeras are more compact and stable than the isolated P450 domains and BMR is a cooperative driver of the unfolding process indicating that denaturation occurs above 35 °C. A higher enthalpy is found for all the transitions in ARO-BMR-5GLY, and the nature of the increased bonds formed in this fusion protein could be ascribed to ionic and electrostatic forces triggered by the domain–domain interactions. This is in line with the results obtained from CO-binding assays, where the highest effect on heme properties was found in the construct ARO-BMR-5GLY that is the one with more interactions within the complex (data not shown). With the analysis of the surface charge distribution for 3A4, the FMN domain of BMR and aromatase (Figure S4) indicates that aromatase has even more positive charges on the distal heme site compared to 3A4, therefore protein–protein interaction within ARO-BMR should be facilitated and even enhanced when compared to 3A4-BMR. Furthermore, the presence of a larger patch of charges is fully in line with the DSC data where we have detected larger enthalpic contributions for both HEME and FMN domains as a function of length of the loop (Table 2), supporting a previously published hypothesis that the addition of five glycines to the connecting loop facilitates the docking of the HEME and FMN domain due to the longer length of the loop and secondary structure loss. The stronger HEME–FMN domain interaction (Figure S4, Table 2) does not work in favor of the conformational restructuring required for an optimal conformational selection process. We have also performed a separate series of experiments in DSC to understand if substrate addition (Figure S5) to ARO-BMR, ARO-BMR-3GLY, and ARO-BMR-5GLY could exert the same effects previously reported for the isolated aromatase as reported in [47]. The data in the case of aromatase chimeras clearly indicate that the expected conformational restructuring seen for aromatase, leading to improved cooperativity of the unfolding process due to a better-defined structure, is absent when substrate is added to aromatase chimeras. Therefore, it can be stated with even more gravitas that the absence of specific conformational restructuring is most probably one of the main reasons behind the observed poor activities of the ARO-BMR systems. All these data are also confirmed for the well-studied 3A4-BMR chimeras [32,33,34,35,53]. In this case, the peak assigned to the FMN domain of BMR does not seem to be stabilized by a longer loop (Figure 4A–F). Thus, since 3A4-BMR is a good model for studying the electron transfer and the activity of CYP3A4 and in the past the molecular Lego was employed for many other CYPs, why did this approach fail in the case of ARO-BMR? How are flexibility, stability, and activity interconnected? In order to answer this question, we need to provide a comprehensive interpretation model (Figure 5).

Substrate binding experiments studied by UV–vis spectroscopy using androstenedione showed poor substrate binding by the three chimeras (Figure S6) when compared to aromatase, where an almost full low-to-high spin transition was obtained in the same conditions [43]. Previous stopped-flow experiments have demonstrated a direct effect of CPR on the affinity of aromatase for the substrate [54]. Moreover, the enthalpic component involved in binding measured in the isothermal titration experiment is more pronounced in the case of the substrate free compared to the substrate bound form of the enzyme, indicating that, without the substrate, more interactions between the two partners (aromatase and CPR) are formed suggesting also conformational changes upon substrate binding [47,54,55,56]. In addition to the effector role of CPR, aromatase was also found to follow a conformational selection process [54,57] for substrate binding that is a well-known substrate binding process for P450s [58,59]. As shown in Figure 5, in a conformational selection model, we can assume that aromatase is present as a heterogeneous population of different conformational states before binding the substrate. In ARO-BMR chimeras, the BMR cannot exert an effector role similar to the one of CPR. Therefore, the question is whether this is due to possible constraints that limit the degrees of freedom of the P450 domain in the chimeric constructs or to a non-productive interaction between ARO and BMR that do not trigger the same conformational effect as CPR. To answer this question, aromatase activity was also measured in the presence of BMR as a separate protein and, also in this case, the activity was very poor (<0.20 pmol min^−1^). This experiment demonstrates that ARO needs a specific redox partner for catalysis, confirming a possible non-productive interaction for the complex.

Additionally, in the case of CYP3A4 a conformational selection process is known to occur for substrate binding [52], but no direct evidence of CPR effector role has been reported yet. Again, BMR can represent a physical constraint that limits the freedom of the heme domain to move and sample alternative conformations. Nevertheless, CYP3A4 is known to for its flexibility, plasticity, and promiscuity in substrate recognition [60,61,62,63] that can compensate for a non-productive interaction within 3A4-BMR chimera. Therefore, substrate binding kinetics are only marginally affected and loop engineering, providing more flexibility to the system, ameliorates electron transfer and coupling.

In order to recapitulate and provide adequate contextualization for our data, we can refer to the relationship between the cytochrome P450 reductase (CPR) dynamics, its reactions cycle [64], and the events required for catalysis to occur. Two models have been proposed for CPR dynamics linked to catalysis. In the first model CPR moves from a more ‘open’ state to a ‘closed’ state when reduced with one equivalent of NADPH and upon reduction by a second NADPH equivalent, CPR is predominantly in an ‘open’ state compatible with electron transfer to P450 [64]. In the second model, the enzyme is ‘closed’ in the oxidized coenzyme free state and upon reduction with NADPH, CPR opens, and electrons can be passed to P450 [64].

Now, if we focus on the key events required for a P450-BMR chimera to function, they can be summarized as follows:

1. The transfer of electrons from NADPH to FMN via FAD reduction within the BMR domain.
2. The transfer of electrons from FMN to the heme domain involving the formation of a functional P450-BMR electron-transfer complex.
3. The binding of the substrate to the heme domain to form a catalytically competent complex.

In this work, we have demonstrated experimentally that the lack of activity of ARO-BMR is not due to due an altered functionality of the ARO-BMR in transferring electrons from NADPH to FAD first and finally to FMN (event 1). Our data indicate that there are evident differences in the way aromatase binds the substrate when the enzyme is in its natural context, a single domain protein, or when artificially fused to the bacterial BMR domain. As already stated above in Figure 5, we provide a comprehensive model to explain the lack of activity of ARO-BMR by suggesting the inability of the chimera to form a catalytically competent complex due to the constraints introduced by BMR to the required conformational selection process for optimal substrate binding in aromatase (event 3). Nevertheless, our data cannot completely rule out the possibility that the obstructed mobility of ARO-BMR given by the creation of an artificial fusion protein may also strongly limit and prevent the conformational opening of BMR, which is necessary for forming an electron-transfer complex of the FMN domain with the P450 [64] (event 2).

## 4. Materials and Methods

### 4.1. Materials

Restriction endonucleases were purchased from New England Biolabs (Ipswich, MA, United States). T4 ligase waspurchased from Promega (Madison, WI, United States). All the media and chemicals were purchased from Merck (Darmstadt, Germany) and Carlo Erba Reagents (Milan, Italy). Estrone ELISA kit was purchased from Diagnostics Biochem Canada (London, ON, Canada). Primers were purchased from Eurofins Genomics (Munich, Germany). Plasmid mini-prep kits were purchased from Merck (Darmstadt, Germany).

### 4.2. Cloning of the BMR Domain of CYP450 BM3

The plasmid coding for CYP450 BM3 was available in our laboratories [65]. BMR reductase domain was sub-cloned between HindIII and NdeI restriction sites in a pCW-Ori(+) vector. The PCR was performed with GGAATTCCATATGCCTAGGTCAAAAAAGGCAGAAAACGC as forward primer and CCCAAGCTTTTACCCAGCCCACACGTC as reverse primer. The clone was sequenced (Eurofins Genomics service) with two commercial primers (both reverse) provided to the company: BMR_Rev_INT (5′-GGATCTTGAAGCTCCACGTATTG) and pCW-INT (5′- GGCCCTTTCGTCTTCAAGCAGA). The digested vector and the gene insert DNA were added to a ligation mixture using T4 DNA ligase A mass of 20–70 ng of vector DNA was used for each reaction screening with various stoichiometric ratios of insert DNA between 1:1 and 1:5 across multiple reactions to maximize the chance of a successful ligation. The digested vector and the gene insert stock solutions were diluted so that additions were of around 1 μL in volume. Ligation reactions were performed at 16 °C temperature for 16 h. Aliquots (5 μL) of each reaction was then used to transform DH5α competent cells which were plated out onto LB agar plates containing ampicillin (100 μg/mL) which were incubated overnight at 37 °C. A single colony from the streak plate was picked and inoculated into 5 mL of sterile LB growth medium in 50 mL a falcon tube and grown at 37 °C, 220 rpm. Cells were centrifuged the day after, and the pellet was used for purification using a miniprep-kit (Merck). Clones were confirmed by DNA sequencing.

### 4.3. Expression and Purification of BMR

The pCW-Ori(+)-BMR vector was transformed into *E. coli* DH5α cells. Overnight cultures (5 mL) were grown O/N at 37 °C, 220 rpm in LB broth supplemented with ampicillin (100 mg/mL), and 1% glucose to prevent autoinduction. The liquid culture was used to inoculate (1:100) 0.5 L of LB in a 2L flask supplemented with ampicillin and the cells were grown at 37 °C, 200 rpm to an OD600 between 0.6 and 0.8 when riboflavin (25 μg/mL) was added. Protein expression was induced by adding 1.0 mM isopropyl-b-D-thiogalactoopyranoside (IPTG). After induction, the culture was cooled down to 28 °C and grown for 18 h (180 rpm). The cells were harvested by centrifugation (20 min, 4000× *g*, 4 °C) and frozen at −20 °C. The purification was started with a cell pellet obtained from 1L culture (LB). It was resuspended in 100 mM Tris-Acetate pH 7.6, 0.5 mM Sucrose, 1 mM EDTA, Lysozyme 0.5 mg/mL + 0.1 EDTA to create sheroplasts and then centrifuged at 4000× *g* for 20 min. The resulted pellet was again resuspended in 100 mM KPi pH 7.6, 6 mM Mg(CH3COO)2, 0.1 mM DTT, 20% glycerol, 0.2 mM PMSF, 21 U/mL DNAse, and extracted by sonication. After ultracentrifugation (41,000× *g*, 45 min) the final pellet was resuspended in 20 mM KPi pH 7.6, 20% Glycerol, 0.1 mM EDTA, 1 mM PMSF, 0.2% NaCholate, 0.2% Triton and ultracentrifuged again 2: (40,000× *g* 1 h). The final supernatant was loaded onto an ADP-sepharose column. After three extensive washes with binding buffer, 100 mM KPi pH 7.6, 20% glycerol, 0.1 mM EDTA, 0.2% Na Cholate, 250 mM NaCl, and finally 100 mM KPi pH 7.6, 20% glycerol, 0.1 mM EDTA, 0.2% Na Cholate, 2 mM Adenosine, the protein was eluted with a 0.2−1 mM NADP+ gradient.

### 4.4. Engineering of ARO-BMR Chimeras

The strategy used in the construction of the human aromatase-BMR (ARO-BMR) chimeras is based on the insertion of 3 or 5 glycines in the pre-existent linker connecting the CYP3A4 domain and the reductase BMR, Pro-Ser-Arg, by using the vector pCW-3A4BMR as a starting template available in our laboratory [32]. Glycine was chosen to allow the highest flexibility of the loop and to support conformational rearrangements between the two domains as much as possible [34,35]. The ARO-BMR chimeras were engineered to contain the N-terminally modified human P450 CYP19A1 (and the BMR reductase domain (aa 477–1054) using the plasmid containing 3A4BMR with the P494RS496, P494RSGGG499, or P494RSGGGGG500 linkers. GCATCTCAAGCATATGGCTAAGAAAACCAGCTCTAAAGGC and CGTAAGCCTAGGGTGTTCCAGACACCTGTC were used as the forward and the reverse primers to amplify by PCR the aromatase gene. The ligation reaction, see Section 4.2, was performed using the aromatase gene insert previously PCR amplified between AvrII and NdeI sticky ends, and the pCW-Ori+ vector harboring the PRS, PRSGGG, or PRSGGGGG—BMR construct (digested with NdeI and AvrII for eliminating 3A4 gene). The control digestion was performed with BamHI and HindIII. BamHI cuts the plasmid twice: one inside and one upstream the aromatase sequence (three fragments are generated 500 bp, 2700 bp, and 5000 bp). HindIII cuts downstream the BMR sequence. Positive clones were sequenced.

### 4.5. Expression and Purification of Aromatase, CYP3A4, ARO-BMR, and 3A4-BMR Chimeras

Expression and purification of aromatase and CYP3A4 was carried out as previously reported [37,47,53]. Heterologous expression of ARO-BMR, ARO-BMR-3GLY, and ARO-BMR-5GLY was performed by transforming competent *E. coli* DH5α cells with the corresponding plasmid vectors. Expression, purification and enzymatic activity of the holoenzyme (120.5 kDa) were achieved according to the methods previously described [32,35]. The proteins were purified using diethylaminoethyl and hydroxylapatite columns as described for 3A4BMR chimeras. The purity of the enzymes was checked by sodium dodecyl sulfate polyacrylamide gel electrophoresis on 10% gel using a Mini-Protean apparatus (Bio-Rad, Hercules, CA, USA) with Coomassie blue staining. Protein concentrations were determined from the absorption at 450 nm of the carbon monoxide bound reduced form by using the Omura and Sato method [66] using an extinction coefficient of 91 mM^−1^ cm^−1^. Protein was reduced with saturating sodium dithionite, and the CO-complex was obtained after one minute of bubbling with carbon monoxide Figure S7.

### 4.6. Rapid Kinetics

Stopped-flow spectrophotometric studies were performed with a Hi-Tech Scientific Co. (Jaipur, India). SF-61DX2 stopped-flow spectrophotometer. All kinetic experiments were performed at 4 °C in anaerobic in filtered 50 mM KPi buffer pH 8.0, as previously reported [10]. The different flavin intermediate species were monitored at 380 nm (anionic red semiquinone), 456 nm (overall reduction), 510 nm (to distinguish the overall reduction from semi-reduction since both anionic and neutral semiquinone are isosbestic at this wavelength), 550 nm (neutral semiquione), and 750 nm (charge transfer complex).

### 4.7. Substrate Binding and Aromatase Activity Assay

Binding of the substrate androstenedione was monitored spectroscopically as a shift of the Soret peak from 418 nm to 394 nm. Increasing concentrations of androstenedione (from 0.5 µM to 10 µM) were added to the ARO-BMR chimeras in 50 mM KPi pH 7.4, 10% glycerol, 200 mM KCl.

For activity assay, reactions were set up using 1 μM ARO-BMR, 0–10 μM androstenedione and 0.5 mM NADPH in a 50 mM KPi pH 7 buffer containing 1 mM DTT, 200 mM KCl [44,45]. Reactions were carried out for 30 min at 30 °C, heat inactivated for 10 min at 90 °C and centrifuged for 5 min at 11,000× *g*. ELISA (competitive ELISA Kit, Diagnostic Biochem Canada Inc.) was performed on the supernatant according to manufacturer instructions in order to detect the quantity of estrone produced by the reaction. Estrone concentration was extrapolated from a calibration curve purchased with the kit. Reactions were also performed in different ionic strength conditions, increasing the concentration of KPi and maintaining the same pH.

### 4.8. Differential Scanning Calorimetry

Differential scanning calorimetry (DSC) was carried out on a Microcal VP-DSC instrument from Malvern Panalytical Ltd (Malvern, United Kingdom) with the following set-up: 25–90 °C temperature gradient, 90 °C/h scan rate, 10 min pre-scan equilibration [67,68]. A solution of 0.6 mg/mL of enzyme was incubated in suitable buffer: 50 mM KPi, 200 mM KCl, 10% glycerol pH 7.0 for ARO constructs; 50 mM KPi, 200 mM KCl, 10% glycerol pH 8.0 for 3A4 constructs. All experiments were conducted with a dilution factor of the storage buffer of 10^6^ of the storage buffer. The denaturation temperature Tm and the ΔH was determined by fitting the data with the ORIGIN embedded program suite. In order to perform the analysis at different scan rates, the cells were pre-equilibrated with at least 10 cycles of buffer in the same setup of the experiment. Data analysis was performed using Microcal Origin software (Malvern, United Kingdom). Cycles of cooling and reheating of the samples were performed to obtain the background for buffer subtraction or test hypothetical refolding. Replicate runs did not vary more than 0.25 °C. None of the constructs exhibited even partial re-folding upon cooling and re-heating the sample indicating full irreversibility of the unfolding processes.

## 5. Conclusions

We have demonstrated that, when using the molecular Lego approach, the chimeras of P450-BMR work well when the P450 domain can compensate with their own internal flexibility and plasticity for the physical constraint given by the presence of an extra polypeptide chain as in the case of 3A4BMR. Careful attention should be paid in the design of P450-BMR chimeras because the functionality of the P450 domain can either benefit (3A4) or be impaired (aromatase) by the BMR fusions depending on intrinsic biochemical features of the P450 domain. Future work should aim to characterize the conformational dynamics of ARO-BMR and 3A4-BMR with other biophysical techniques that can shed light on possible blocking of opening–closing transitions necessary for the interdomain electron transfer [69] or the ability of the BMR domain to oligomerize in relation to catalysis [70]. Moreover, the structural and functional characterization of the chimeras in the absence or presence of known physiological partners, such as cytochrome b5, should also be taken into consideration [69].

## Figures and Tables

**Table 1:** Effect of BMR fusion on aromatase and P450 3A4 activity.

Enzyme	k_cat_ (min^−1^)	Enzyme	k_cat_ (min^−1^)	Refs.
ARO	1.9 ± 0.1	CYP3A4	2.74 ± 0.26	[44,46]
ARO-BMR	0.0021 ± 0.0002	3A4-BMR	0.120 ± 0.004	[35]
ARO-BMR-3GLY	0.0023 ± 0.0001	3A4-BMR-3GLY	0.156 ± 0.003	[35]
ARO-BMR-5GLY	0.0026 ± 0.0002	3A4-BMR-5GLY	0.216 ± 0.007	[35]
Activity measurements are based on estrone formation for ARO and ARO chimeras and 6β-hydroxylation of testosterone for 3A4 and 3A4 chimeras.				

**Table 2:** ARO-BMR and 3A4-BMR chimeras melting temperature (T_m_) values and enthalpies (ΔHcal). The standard error of mean is not reported in Table 2 because it is below 1.5% of each of the reported data.

	T_m_ Melting T/°C			ΔH kcal/mol/°C		
	HEME DOMAIN	FAD DOMAIN	FMN DOMAIN	HEME DOMAIN	FAD DOMAIN	FMN DOMAIN
ARO-BMR	45.66	49.48	56.37	181.14	324.89	33.97
ARO-BMR-3GLY	44.08	48.54	55.18	146.75	390.04	84.76
ARO-BMR-5GLY	46.23	49.61	56.36	464.29	549.98	324.47
3A4-BMR	44.38	48.47	54.04	67.52	266.94	109.35
3A4-BMR-3GLY	45.25	48.78	54.70	99.97	239.57	95.76
3A4-BMR-5GLY	45.71	49.18	54.50	143.01	438.18	90.48

## Supplementary Materials

The following supporting information can be downloaded at: https://www.mdpi.com/article/10.3390/ijms23073618/s1.
