# Supplementary material for: Molecular Lego of Human Cytochrome P450: The Key Role of Heme Domain Flexibility for the Activity of the Chimeric Proteins
Source: Int J Mol Sci. 2022 Mar 25;23(7):3618. doi: 10.3390/ijms23073618 (PMC8998974; doi:10.3390/ijms23073618)
Supplement: Supplementary file 1 [file ijms-23-03618-s001.zip › ijms-1551650-supplementary.pdf]

A) B)

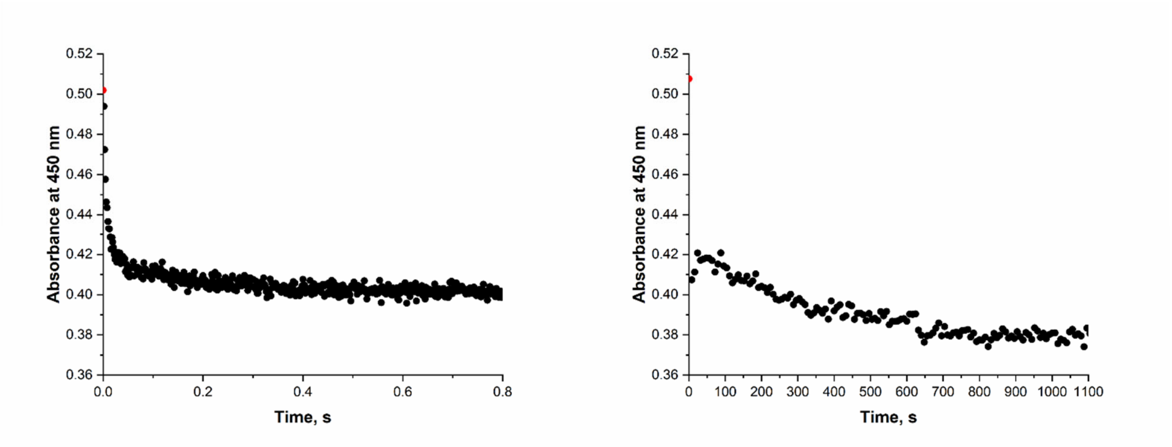

Figure S1 Kinetics of ARO-BMR reduction shown as the change in absorbance at 450 nm versus time to monitor the formation of the ARO-BMR-CO complex formation obtained by mixing a solution of NADPH and a solution of CO saturated protein in a 10:1 ratio. A) Absorbance change in 0.8 seconds, B) absorbance change in 1100 seconds. All datapoints are shown in black except the first one shown in red. No ARO-BMR-CO complex could be detected even after 1100 seconds.

A) B)

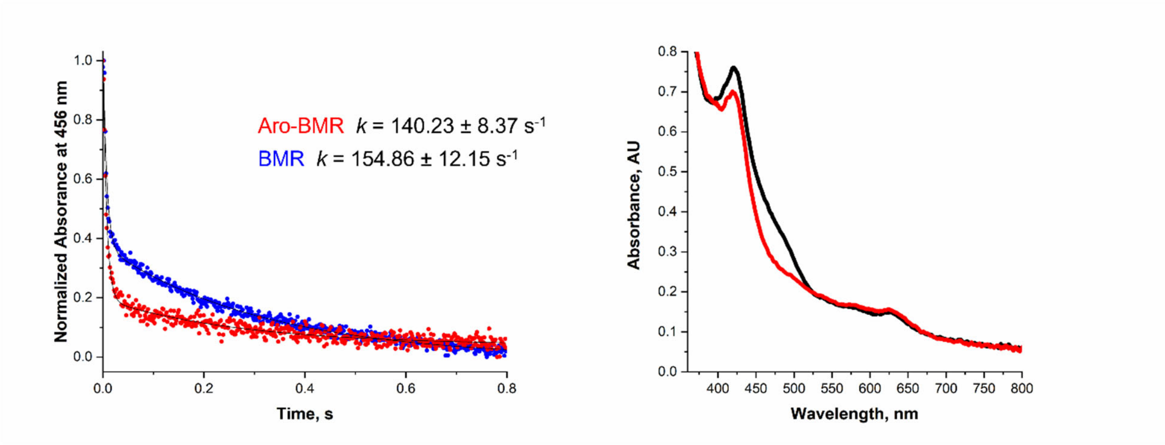

Figure S2 Kinetics of flavin reduction for ARO-BMR (red points) and BMR (blue points) obtained by mixing a solution of NADPH and a solution of protein in a 10:1 ratio. A) Absorbance change in 0.8 seconds, B) First (black) and last (red) spectrum recorded in a 0.8 seconds timespan for ARO-BMR.

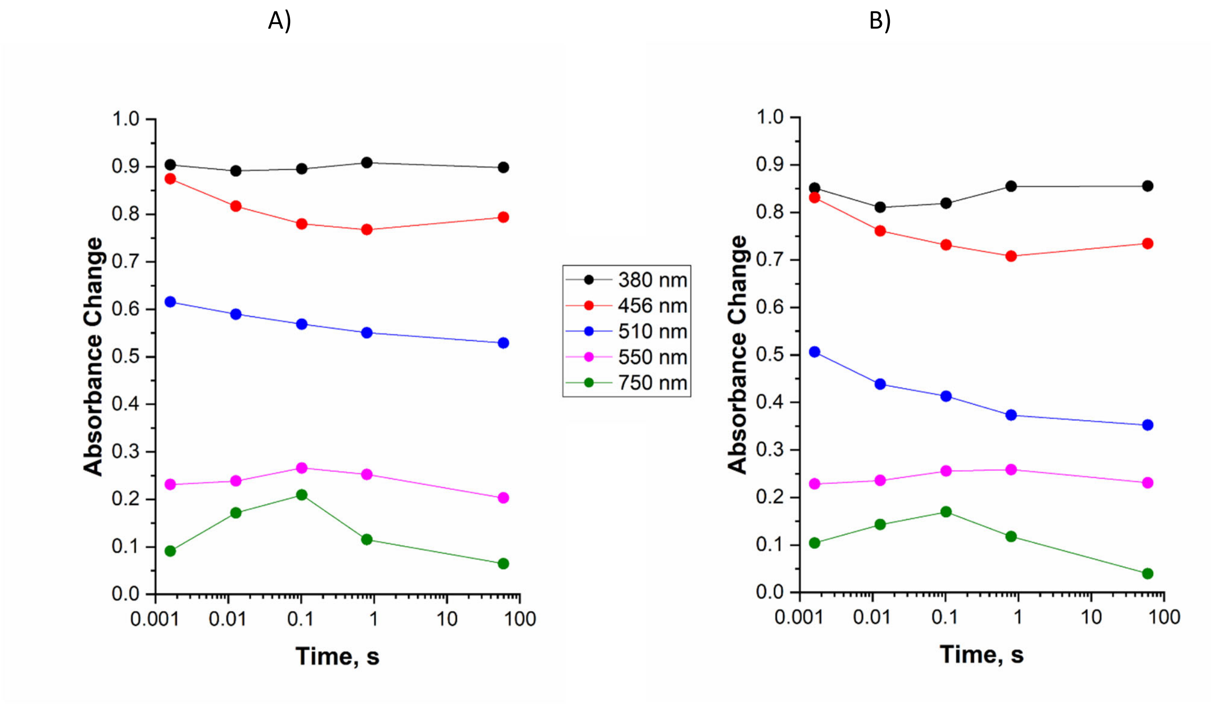

Figure S3 Kinetic absorbance changes during the reduction of A) ARO-BMR or B) BMR by a 1:1 ratio of NADPH to BMR. BMR in 50 mM KPi buffer, pH 8.0, was mixed with an equal volume of NADPH in the stopped-flow spectrophotometer. For ARO-BMR: The data were offset by 0.3 and multiplied by 1 at 380 nm, offset by 0.4 and multiplied by 1 at 456 nm, multiplied by 5 and offset by -0.5 at 510 nm, multiplied by 5 with a -0.12 offset at 550 nm, and multiplied by 10 with a -0.05 offset at 750 nm. For BMR: The data were offset by 0.55 and multiplied by 1 at 380 nm, offset by 0.55 and multiplied by 1 at 456 nm, multiplied by 5 and offset by -0.1 at 510 nm, multiplied by 5 with no offset at 550 nm, and multiplied by 10 with a -0.005 offset at 750 nm.

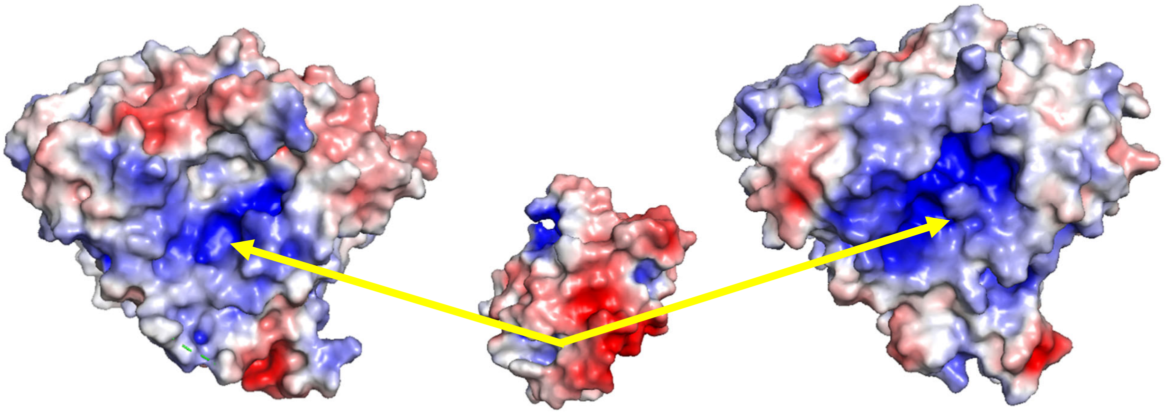

Figure S4 Surface charge distribution of P450 3A4 (left, PDB ID 1TQN) and the FMN binding domain of P450 BM3 (center, PDB ID 1BVY) and aromatase (right, PDB ID 4KQ8).

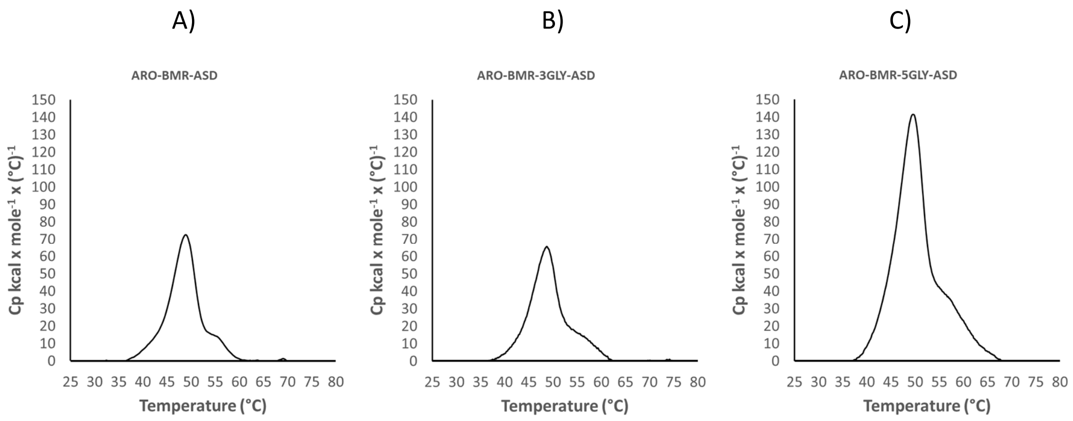

Figure S5 Thermograms of (A) ARO-BMR, (B) ARO-BMR-3GLY and (C) ARO-BMR-5GLY in the presence of 10 µM androstenedione (ASD).

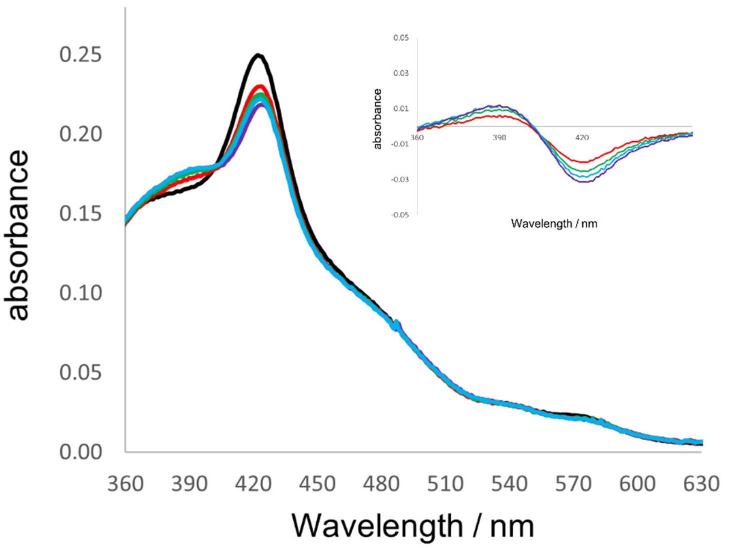

Figure S6 Typical UV–vis absorption spectra of ARO-BMR titrated with androstenedione where the inset indicates the difference spectra. The substrate concentration is 0.19 µM (red trace), 0.38 µM (green trace), 0.56 µM (cyan trace) and 0.75 µM (violet trace).

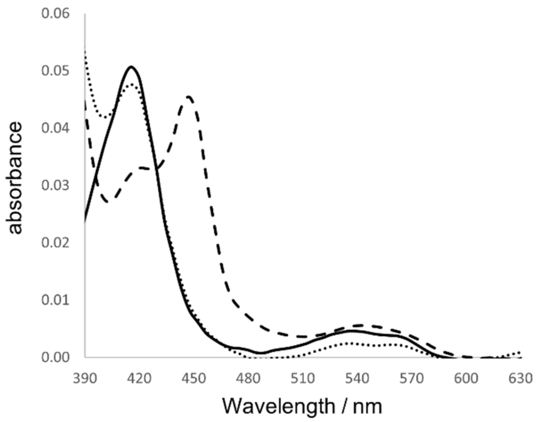

Figure S7 CO binding CO binding assay of aromatase. Oxidised, reduced and CO bound spectra are shown in solid, dotted and dashed lines respectively.
